# Assessing Detection Accuracy of Computerized Sonographic Features and Computer-Assisted Reading Performance in Differentiating Thyroid Cancers

**DOI:** 10.3390/biomedicines10071513

**Published:** 2022-06-26

**Authors:** Hao-Chih Tai, Kuen-Yuan Chen, Ming-Hsun Wu, King-Jen Chang, Chiung-Nien Chen, Argon Chen

**Affiliations:** 1 Department of Surgery, National Taiwan University Hospital and College of Medicine, Taipei 100225, Taiwan; taihc1@ntu.edu.tw (H.-C.T.); dtsurg51@ntuh.gov.tw (K.-Y.C.); 010028@ntuh.gov.tw (M.-H.W.); kingjen@ntu.edu.tw (K.-J.C.); 2 Graduate Institute of Industrial Engineering, National Taiwan University, Taipei 106216, Taiwan

**Keywords:** thyroid nodules, sonographic features, computer-assisted detection, MRMC study

## Abstract

For ultrasound imaging of thyroid nodules, medical guidelines are all based on findings of sonographic features to provide clinicians management recommendations. Due to the recent development of artificial intelligence and machine learning (AI/ML) technologies, there have been computer-assisted detection (CAD) software devices available for clinical use to detect and quantify the sonographic features of thyroid nodules. This study is to validate the accuracy of the computerized sonographic features (CSF) by a CAD software device, namely, AmCAD-UT, and then to assess how the reading performance of clinicians (readers) can be improved providing the computerized features. The feature detection accuracy is tested against the ground truth established by a panel of thyroid specialists and a multiple-reader multiple-case (MRMC) study is performed to assess the sequential reading performance with the assistance of the CSF. Five computerized features, including anechoic area, hyperechoic foci, hypoechoic pattern, heterogeneous texture, and indistinct margin, were tested, with AUCs ranging from 0.888~0.946, 0.825~0.913, 0.812~0.847, 0.627~0.77, and 0.676~0.766, respectively. With the five CSFs, the sequential reading performance of 18 clinicians is found significantly improved, with the AUC increasing from 0.720 without CSF to 0.776 with CSF. Our studies show that the computerized features are consistent with the clinicians’ findings and provide additional value in assisting sonographic diagnosis.

## 1. Introduction

Thyroid cancer is the most common endocrine cancer, and its incidence has increased dramatically by an average of 4.5% annually [1]. Accurate identification of thyroid cancer is crucial for effective treatment. Ultrasonography is the most common tool for early detection of thyroid cancer because it is readily available and noninvasive. In the past decade, use of high-resolution ultrasound has resulted in improved detection of thyroid nodules [2,3]. Nevertheless, most of the nodules are benign, and thyroid cancers only account for 7–15% of detected nodules [4]. Identification of malignant nodules is critical to avoid unnecessary fine-needle aspiration (FNA) biopsy and surgical procedures. Medical guidelines, most notably the management guidelines by the American Thyroid Association (ATA) [4] and Thyroid Imaging Reporting and Data System (TI-RADS) by the American College of Radiology (ACR) [5], have been developed and recommended to clinicians on how to identify and use the sonographic features for differentiation of thyroid cancers. Important features include micro-calcifications, hypo-echogenicity, irregular margins, taller-than-wide shape, etc. [4,6,7,8,9,10]. However, presence of the sonographic features is determined based on a physician’s subjective interpretation, which may be influenced by education and experience. Interpretation discrepancies among clinicians or even by the same clinicians at different times have become a major issue that hinders the diagnosis and treatment of thyroid nodules [11,12].

Recent development of AI/ML technologies has given rise to the use of CAD software devices in clinical practice [13,14], to assist clinicians with improving their diagnosis accuracy and workflow effectiveness. The CAD solutions have been applied to ultrasound imaging of various diseases, such as breast cancer [15,16] and liver lesions [17]. One CAD software device has emerged to give a second opinion on image interpretation [18], to reduce inter-observer variation in breast images [16,19]. Studies have also reported applications of CAD devices to ultrasound imaging of thyroid nodules [20,21,22,23,24,25,26,27,28]. In particular, the effectiveness of computerized sonographic features (CSF) to differentiate malignant nodules has been demonstrated [20,21,22,23,24]. The CSFs provided by a CAD device are used to assist clinicians in interpreting the images and then making recommendations based on the clinicians’ professional judgment and/or medical guidelines. Although both clinicians’ finding and computerization of sonographic features are shown helpful in thyroid cancer diagnosis, the computerized sonographic features have not yet been validated by comparing to the clinicians’ sonographic feature findings and by studying their effect in assisting clinicians’ reading of the thyroid sonograms.

**Figure 1:**
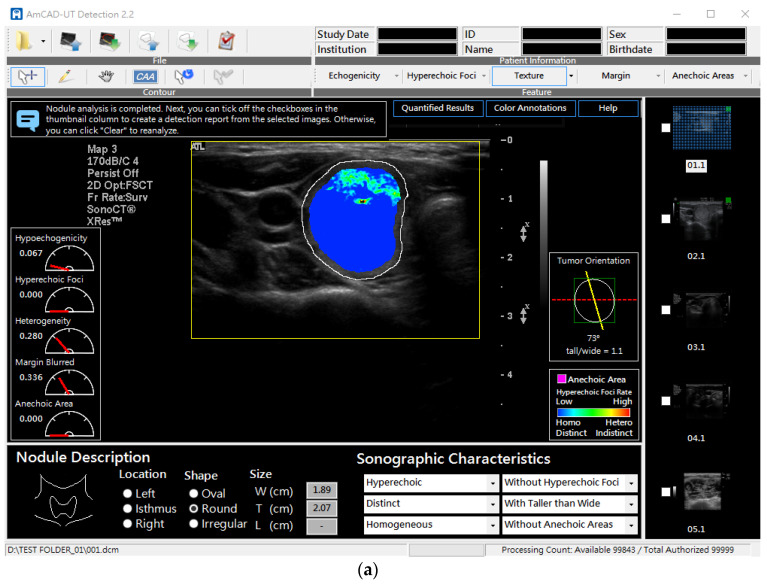

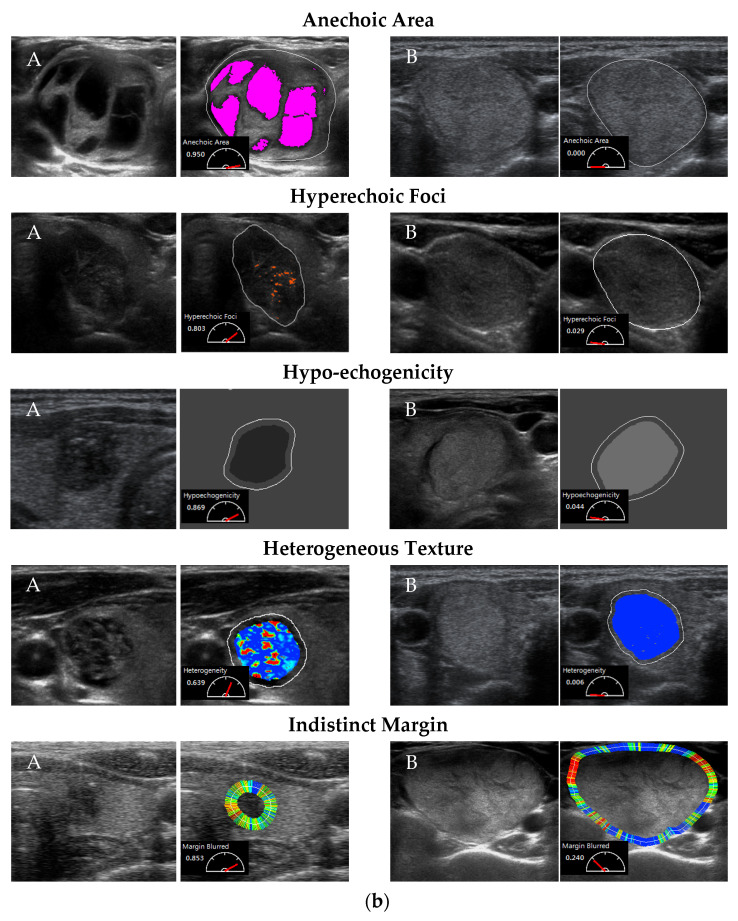
AmCAD-UT: (**a**) Graphical user interface; (**b**) A: nodules with features present; B: nodules without the features. Features are visualized, with colors indicating the severity or likelihood of the feature presence—the warmer the color the more likely or severer the feature’s presence. Quantified values of the computerized features ranging from 0 to 1 are shown and displayed in the pointer meters.

In this study, an FDA-cleared CAD software device, AmCAD-UT (AmCad BioMed Co., Taipei, Taiwan) (Figure 1a), is employed to validate the software’s detection and quantification of the sonographic features (Figure 1b) against the ground truth established by a panel of thyroid specialists and then to perform an MRMC study to test the reader’s performance sequentially assisted with the computerized features calculated by the CAD device.

## 2. Materials and Methods

### 2.1. Database for Computerized Features Testing

**Figure 2:**
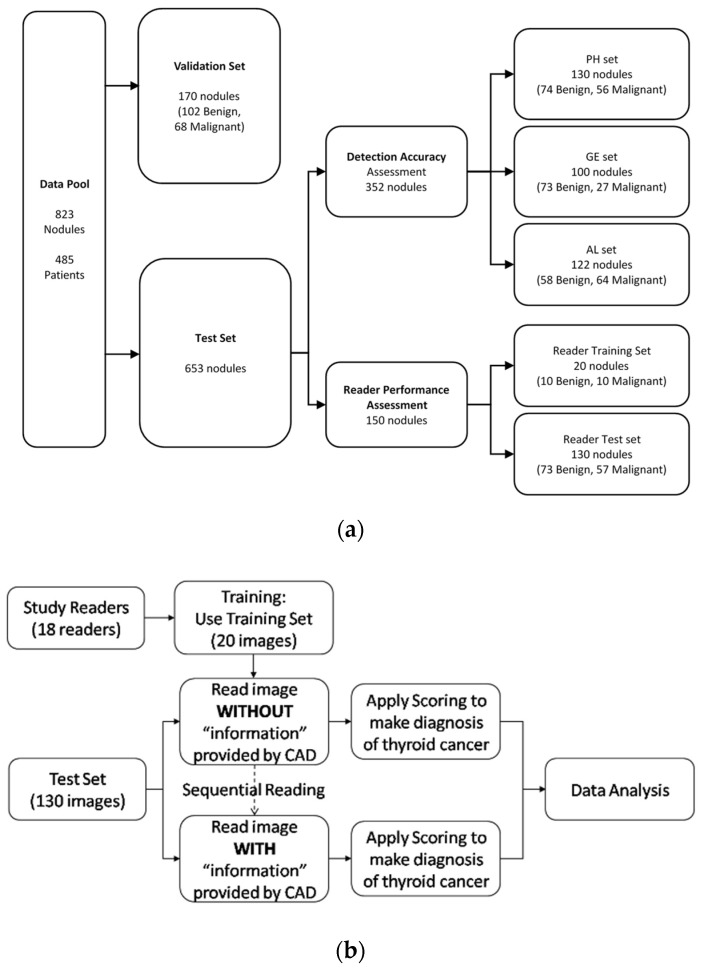
Study datasets: (**a**) Datasets for the validation, detection accuracy test, and reader performance assessment. (**b**) Datasets and readers for the MRMC reader performance study.

The Institutional Review Board of the National Taiwan University Hospital (NTUH) approved this prospective study (200805039R). Informed consent was obtained from all participants and all patient identifiers were removed from the images used in the study. The database consisted of a collection of thyroid sonograms of patients who underwent a thyroidectomy due to suspicious thyroid carcinoma, follicular neoplasm, or symptomatic nodular goiter diagnosed by ultrasound imaging and FNA cytology, at NTUH. Since the quality of the sonograms greatly depended on the ultrasound scanners and might affect both the clinicians’ finding and computerization of the sonographic features, we collected for this study sonograms obtained from different ultrasound scanners. The sonograms were acquired in DICOM format using Philips HDI 5000 (denoted as PH), GE Voluson 730 PRO (denoted as GE), and ALOKA Prosound2 (denoted as AL) ultrasound scanners, with 5–12 MHz linear multifrequency probes under identical imaging setting by certified technicians. The images were ineligible if the nodule size was larger than the width of the probe array or if the single nodule was not separable from another in cases of multinodular goiters. Finally, sonograms of 823 nodules (663 patients) were included in the database (Figure 2a). The major ethnic group was Chinese. Our previous studies investigated the effectiveness of specific computerized sonographic features, namely, calcification, heterogeneity, or echogenicity, in distinguishing malignant from benign nodules [20,21,22,23,24], whereas the current study validate the detection accuracy against the ground truth established by a panel of three thyroid specialists. A set of images was used for algorithm validation, consisting of 170 sonograms (102 benign and 68 malignant). In total, 653 sonograms were used for testing. Of these, 352 sonograms were randomly chosen for test of feature detection accuracy, and another 150 images from the Philips scanner were chosen for reading performance assessment (Table 1a). The pathology results of the nodules and cancer type distribution are shown in Table 1b. Among the 150 sonograms, 20 (10 benign and 10 malignant) were used as the training set for the 18 readers to get familiar with the CAD software user interface and to train themselves using the software to differentiate malignant nodules. The remaining 130 sonograms were used for the MRMC study (Figure 2b).

### 2.2. Testing CSF Detection Accuracy

The AmCAD-UT software device was developed to assist clinicians in analyzing the regions of interest (ROI) on the thyroid sonograms. Figure 1a shows the interface of the AmCAD-UT software. The software produced five computerized sonographic features (CSFs), namely, anechoic areas, hyperechoic foci, hypoechoic patterns, heterogeneous textures, and indistinct margins, providing their quantified values and visualizing them, using colors, to assist clinical differentiation of thyroid nodules. The quantification and visualization algorithms of AmCAD-UT have been disclosed in previous studies [20,21,22,23,24].

To validate the feature detection, we tested the quantified value of each sonographic feature against the ground truth, indicating the presence or absence of each feature on the sonogram (Table 2). The ground truth was determined by a panel of three thyroid specialists. All three specialists, certified to interpret the sonograms, with an average of 8.6 years (range 8–10 years) of experience and an average of 1366 readings (ranging from 700 to 2400 readings), independently read the sonograms to define the ROIs of the nodules and to determine the presence or absence of each feature. In cases of a discrepancy in interpretation, consensus was achieved by discussions among the panelists.

For all sonograms, the CAD device automatically performed detection and quantification of each feature and electronically stored the values in a database for subsequent analysis. The quantified values between nodules with the feature present and those without the feature were compared using a *t*-test and a *p*-value less than 0.05 was considered statistically significant. Since each feature was quantified as a continuous value to indicate a higher likelihood of presence by a larger value of the computerized feature, the receiver operating characteristic (ROC) curve for each feature was also generated againstthe corresponding “ground truth”, with the area under the ROC curves (AUC) calculated to represent the detection accuracy. If the detection accuracy was 100%, i.e., AUC = 1.0, it meant a cut-off point can be found for the CSF to determine the presence of the sonographic feature, such that the findings by the computer and by the specialist panel were in 100% agreement. The higher the AUC value, the higher the agreement between the CSF and the ground truth. The statistical analysis was performed using MedCalc version 10.6.0.0 (MedCalc Software Ltd, Ostend, Belgium).

### 2.3. Assessing Diagnosis Performance of Readers Assisted with CSF

To assess whether the assistance of CSF provided by the CAD device can improve the readers’ diagnosis accuracy, a multiple-reader multiple-case (MRMC) study [29,30,31] was performed. We recruited 18 clinicians (readers) to read 130 thyroid nodule sonograms. All the clinicians were licensed for an average of 8.78 years (ranging from 1 to 25 years experience) to perform ultrasound scans and interpret the sonograms, but had no experience in using AmCAD-UT. No reader had foreknowledge of the corresponding pathology results (benign or malignant) of the thyroid nodules. We first trained the readers with 20 training sonograms and the corresponding pathology results (10 benign or 10 malignant) to get the readers familiarized with the interface of the CAD software. Then, each of the 18 readers read each of 130 sonograms first without the CSF and then sequentially read the sonogram with the CSF provided [30]. The sequential reading with the CSF was to mimic how the CAD device would be used in clinical practice where the CSF information was provided as an integral part of the clinical reading and interpretation of sonograms. In other words, a reader read and scored every sonogram twice—one without CSF and one with CSF—for all 130 images. The order of the 130 sonograms was randomized and different for every reader. The scoring was scaled from 0 to 100 (0 = absolute benign, 100 = absolute malignant). We used the DBM MRMC 2.32 software (based on the Dorfman–Berbaum–Metz method) for generation of the ROC curves based on bi-normal models and for estimates of random effects of readers and cases [29,32,33,34]. Paired ROC curves of readers’ scoring without and with assistance of CSF were generated against pathology, and the paired AUCs calculated. We also performed subgroup analysis for readers with different levels of experience. Readers with more than 6 years’ experience of interpreting sonograms were referred to as senior readers and the others were called junior readers. We calculated the paired AUC for the two groups separately and compared the paired AUCs between the two groups.

## 3. Results

### 3.1. Detection Accuracy of Computerized Sonographic Features

The detection accuracy of each computerized feature was tested against the ground truth (Table 2). Regardless of the ultrasound scanner types, the difference between the two groups of quantified values (with or without presence of anechoic areas, hyperechoic foci, hypoechoic pattern, heterogeneous texture and indistinct margin) were significant, ranging from a *p*-value < 0.0001 to a *p*-value = 0.0347 (Table 3). The lowest agreement (*p*-value = 0.0045~0.0347) was observed in detecting the heterogeneous texture and the highest agreement between the computerized values and panel findings was observed for the anechoic area and the hyperechoic foci detection.

**Figure 3:**
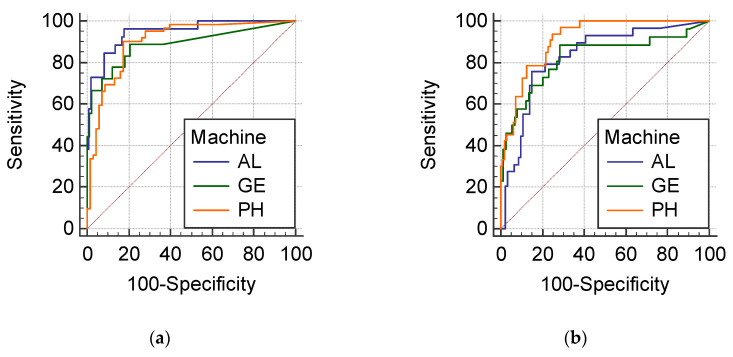

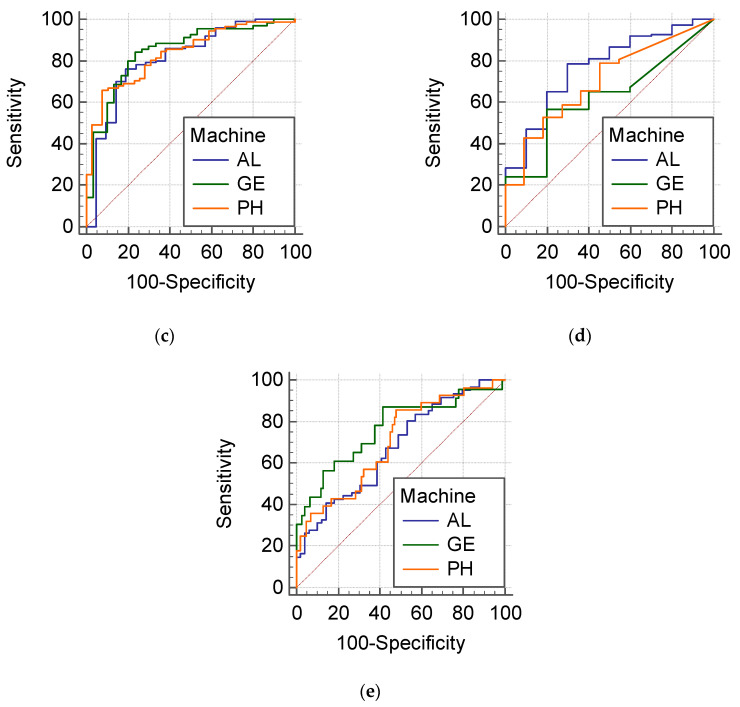
ROC of detecting the presence by the computerized features: (**a**) anechoic area; (**b**) hyperechoic foci; (**c**) hypoechoic pattern; (**d**) heterogeneous texture; (**e**) indistinct margin.

Similarly, in terms of the AUC against the ground truth, the detection accuracies of the quantified anechoic areas, hyperechoic foci, echogenicity (hypoechoic pattern), heterogeneous texture and indistinct margin were 0.946–0.888, 0.825–0.913, 0.812–0.847, 0.627–0.77 and 0.676–0.766 for various ultrasound scanners (Table 3 and Figure 3), respectively. The detection accuracies of the quantified heterogeneous texture and indistinct margin appeared to be lower than that of the quantified anechoic areas, hyperechoic foci, and hypoechoic pattern. These AUCs also indicated good agreement between the computerized feature values and the panel’s readings.

### 3.2. Reader Performance Assisted with CSF

**Figure 4:**
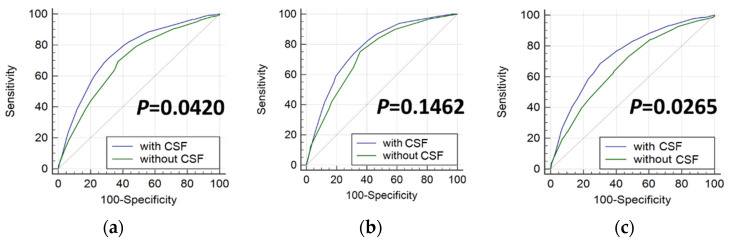
ROC curves with and without CSF: (**a**) all readers; (**b**) senior readers; (**c**) junior readers.

The reader performance using the computerized sonographic features generated by the CAD device was tested against the corresponding pathology. The accuracy of the readers in diagnosing malignant thyroid nodule sonograms was evaluated by the AUC based on the readers’ scoring. AUCs without and with the computerized sonographic features (CSF) for each reader are shown in Table 4a. The mean AUC with CSF was significantly greater (*p*-value = 0.0420) than that without CSF using the DBM MRMC test, as shown in Table 4b. Moreover, the statistics showed that the difference was mainly observed for the junior readers (*p*-value = 0.1462 and 0.0265, respectively, for the senior and junior readers). Figure 4 shows the graph of the paired ROC curves. Differences could be observed between the ROCs with and without CSF for all readers, indicating improvement in the reader’s performance with the assistance of computerized features. In particular, Figure 4c showed the distinct ROC curves for the junior readers. However, only a slight difference was found in Figure 4b between the pair of ROC curves for senior readers. This demonstrated that the computerized features were especially beneficial to junior clinicians to supplement their relatively less experience in making diagnosis. Though an improvement was also observed for the senior readers assisted by the computerized features, a bigger sample size of senior readers might be required to show the significance.

## 4. Discussion

### 4.1. Population and Features

The distribution and percentage of thyroid cancers were similar to that reported internationally [35,36,37], representing actual clinical practice and the demographic distribution of the studied population. Therefore, the possible effect of the nodule types and their distributions should be considered negligible. In addition, because the CSFs were found highly agreeable with the clinicians’ findings for all three types of ultrasound scanners included in this study, the feature computerization of the CAD device should be generalizable to thyroid sonograms acquired for a general patient population in practical clinical situations.

### 4.2. CSF Accuracy

The sonographic features evaluated by the CAD device were tested to be significantly in agreement with the specialist’s judgement (ground truth). The heterogeneous texture feature appeared to be less agreeable and was possibly due to the extremely unbalanced reading results by the specialist panel, where only 5–8.5% of nodules were not deemed to be heterogeneous. In clinical practice, even a slight unsmooth trace within a nodule would lead to a judgement of a heterogeneous nodule. In contrast, the CAD software provided the quantified values evaluating the degree of heterogeneity, which was more functional, with a continuous severity rating. Another less agreeable feature was the indistinct margin. An indistinct margin was supposedly indicated for a possible infiltration by a malignant nodule. However, determination of indistinct margins was highly subjective and greatly influenced by the clarity of the sonogram and the aim angle of the ultrasound probe. The readings of indistinct margins were thus with a relatively high variability and appeared to be less reliable. Since the CSFs were produced by the CAD software independently without interaction with clinicians, they could serve as second opinions to assist clinical diagnosis.

### 4.3. Reader Performance with CSF

An MRMC analysis was necessary to test the performance of a CAD device, since the radiological device was to assist the physicians and had to been shown of value to the physicians in making diagnosis. The MRMC study design offered a comprehensive analysis of the role of the device in actual clinical situations where clinicians of different experiences might use the device for reading a variety of cases [31]. Both the reader variability and interactions between readers and cases were considered by the DBM model in the MRMC analysis [34]. The scoring by rating the malignancy potentials of the nodules reflected the readers’ interpretation confidence without and with the device’s assistance. In this study, a total of 18 readers were recruited for the test and all readers received training by reading 20 sonograms prior to the test. The test sonograms were randomly selected from a sufficiently large database to avoid possible sampling bias. The analysis results demonstrated that the reading performances of clinicians, in terms of AUC, were significantly improved when assisted with the CSFs produced by the CAD. We had also observed that the greatest improvements were mainly made by the junior readers, although the higher diagnosis accuracy, with an average AUC near 0.8, was achieved by the senior readers. The CSFs appeared to be of value to both junior and senior readers, with the accuracy approaching and not exceeding 0.8. Since the improvement made by the senior readers was only about 5% (from AUC = 0.759 to 0.799), a larger number of senior readers might be needed to prove the statistical significance.

### 4.4. CAD Device in Clinical Practice

Major advantages of the CAD device included providing a critical second opinion, reducing time-consuming procedures, and avoiding oversight and interobserver variation [13]. A CAD device could serve as a reliable second opinion because it provides accurate information of the sonographic features in concordance with the ground truth. Independent double reading by another clinician was an alternative way to improve the detection accuracy [38]. However, independent double reading was a labor-demanding work for clinicians and might not be as efficient and effective as the assisted reading of a CAD device [19]. Furthermore, the sonographic reading of a human clinician was greatly dependent on one’s past experience and often subject to momentary feel. In contrast, the CAD device could provide consistent and objective evaluation regardless of the time and environment settings, and thus could further reduce the interobserver variation [39].

Several CAD algorithms had been proposed for thyroid ultrasound images by other authors [25,26,27,28]. Acharya et al. characterized the thyroid nodules into benign and malignant classes using a combination of texture and discrete wavelet transform [25]. Chang et al. used the support vector machine classifier to differentiate malignant and benign nodules and showed accuracy similar to that obtained via visual inspection by radiologists [26]. Choi et al. suggested that the sensitivity of the junior reader was as good as that of the senior readers by using a CAD device with a lower the specificity [27]. Most recently, Wu et al. reported an MRMC study that showed a significant improvement in reading performance with assistance of the CAD device after a washout period [28]. Although the above studies assessed the differentiation of malignant and benign nodules by using the CAD device, this study would be the first study to evaluate the CAD device’s performance in reading the sonographic features through comparison to readings by a panel of specialists and to assess the CSFs’ effects on the reader performance via an MRMC study of sequential readings with and without CSF.

### 4.5. Study Limitation

This study had limitations regarding the sonographic features evaluated. First, hyperechoic foci were not further differentiated into microcalcifications, coarse calcifications, rim-shape calcifications, or colloid [20]. Second, the echogenicity level determined by the panel was not further classified into the four levels, namely, hyperechoic, isoechoic, mildly hypoechoic, and markedly hypoechoic [23], commonly used in clinical practice. Third, though the sequential readings without and with the CSF mimicked how the CAD device would be used in clinical practice but the sequential readings without a washout period might result in a recollection bias and impair the effect of the assistance by the CAD device.

In conclusion, the CAD device visualized and quantified the thyroid sonographic features in high concordance with a specialist panel and was shown to significantly improve clinicians’ reading of the nodule’s malignancy risk.

## Figures and Tables

**Table 1:** Demographics of the patients and nodules.

	**Detection Accuracy Assessment** **PH Set**			**Detection Accuracy Assessment** **GE Set**		
	**Patients** **(*n* = 113)**			**Patients** **(*n* = 84)**		
	**Benign** **(*n* = 74)**	**Malignant** **(*n* = 56)**	**Total (*n* = 130)**	**Benign** **(*n* = 73)**	**Malignant** **(*n* = 27)**	**Total** **(*n* = 100)**
**Gender** No. (%)						
Male	14 (18.9)	9 (16.1)	23 (17.7)	14 (19.2)	3 (11.1)	17 (17.0)
Female	60 (81.1)	47 (83.9)	107 (82.3)	59 (80.8)	24 (88.9)	83 (83.0)
**Age (year)**						
Mean ± SD *	48.2 ± 13.8	48.6 ± 15.4	48.3 ± 14.5	51.1 ± 13.2	50.1 ± 16.6	50.5 ± 14.1
Range	20.9–76.9	11.2–71.6	11.2–76.9	20.9–75.3	20.4–84.8	20.4–84.8
**Size of Nodule (cm)**						
Mean ± SD *	2.38 ± 0.88	1.87 ± 0.86	2.16 ± 0.90	2.50 ± 0.94	1.86 ± 0.72	2.33 ± 0.93
Range	0.94–4.37	0.49–4.09	0.49–4.37	0.71–4.16	0.87–3.66	0.71–4.16
(**a**) Demographics of patients and nodule images acquired from Philips (PH), GE, and Aloka (AL) ultrasound scanners for detection accuracy and reader performance assessment.						
	**Detection Accuracy Assessment** **AL Set**			**Reader** **Performance Assessment**		
	**Patients** **(*n* = 107)**			**Patients** **(*n* = 129)**		
	**Benign** **(*n* = 58)**	**Malignant** **(*n* = 64)**	**Total** **(*n* = 122)**	**Benign** **(*n* = 83)**	**Malignant** **(*n* = 67)**	**Total** **(*n* = 150)**
**Gender** No. (%)						
Male	8 (13.8)	14 (21.9)	22 (18.0)	16 (19.3)	11 (16.4)	27 (18.0)
Female	50 (86.2)	50 (78.1)	100 (82.0)	67 (80.7)	56 (83.6)	123 (82.0)
**Age (year)**						
Mean ± SD *	45.9 ± 9.5	42.7 ± 11.8	44.2 ± 10.8	48.5 ± 13.9	47.8 ± 14.8	48.2 ± 14.3
Range	21.7–64.3	1.5–74.0	1.5–74.0	20.9–76.9	11.2–71.6	11.2–76.9
**Size of Nodule (cm)**						
Mean ± SD *	2.45 ± 1.06	1.48 ± 0.72	1.94 ± 1.02	2.38 ± 0.86	1.86 ± 0.83	2.15 ± 0.88
Range	0.53–4.29	0.53–4.12	0.53–4.29	0.94–4.37	0.49–4.09	0.49–4.37
(**b**) The pathology diagnosis results of the 823 nodules.						
	**Pathology of Nodules**			**No. (%)**		
**Benign** (*n* = 499)	Nodular hyperplasia			428 (85.8)		
	Follicular adenoma			70 (14.0)		
	Unidentified adenoma			1 (0.2)		
**Malignant** (*n* = 324)	Papillary thyroid carcinoma			296 (91.4)		
	Follicular thyroid carcinoma			15 (4.6)		
	Medullary thyroid carcinoma			5 (1.5)		
	Anaplastic carcinoma			5 (1.5)		
	Others			3 (1.0)		

* SD = standard deviation.

**Table 2:** Ground truth established by the panel of thyroid ultrasound specialists.

Sonographic Features	Determined by a Panel of Specialists	PH No. (*%*)	GE No. (%)	AL No. (%)
Anechoic Areas	Absence	68 (52.3%)	82 (82.0%)	96 (78.7%)
	Presence	62 (47.7%)	18 (18.0%)	26 (21.3%)
Hyperechoic Foci	Absence	97 (74.6%)	74 (74.0%)	93 (76.2%)
	Presence	33 (25.4%)	26 (26.0%)	29 (23.8%)
Hypoechoic Pattern	Absence	39 (30.0%)	30 (30.0%)	21 (17.2%)
	Presence	91 (70.0%)	70 (70.0%)	101 (82.8)
Heterogeneous Texture	Absence	11 (8.5%)	5 (5.0%)	10 (8.2%)
	Presence	119 (91.5%)	95 (95.0%)	112 (91.8%)
Indistinct Margin	Absence	102 (78.5%)	77 (77.0%)	49 (44.5%)
	Presence	28 (21.5%)	23 (23.0%)	61 (55.5%)

**Table 3:** Test results of the computerized sonographic features’ detection accuracy.

		Anechoic Areas	Hyperechoic Foci	Hypoechoic Pattern	Heterogeneous Texture	Indistinct Margin
**PH** (*n* = 130)	AUC (95% CI)	0.902 (0.838–0.947)	0.913 (0.850–0.955)	0.837 (0.762–0.896)	0.701 (0.614–0.778)	0.702 (0.616–0.779)
	*p*-value	<0.0001	<0.0001	<0.0001	0.0347	0.0007
**GE** (*n* = 100)	AUC (95% CI)	0.888 (0.809–0.942)	0.825 (0.736–0.894)	0.847 (0.761–0.911)	0.627 (0.525–0.722)	0.766 (0.670–0.845)
	*p*-value	0.0001	<0.0001	<0.0001	0.0323	0.0002
**AL** (*n* = 122, *n* = 110 for Margin)	AUC (95% CI)	0.946 (0.890–0.979)	0.830 (0.751–0.892)	0.812 (0.732–0.877)	0.77 (0.685–0.841)	0.676 (0.580–0.762)
	*p*-value	<0.0001	0.0002	0.0156	0.0045	0.0002

PH: Philips HDI 5000; GE: GE Voluson 730; AL: ALOKA Prosound2.

**Table 4:** Reader performance in terms of AUC without and with CSF: (**a**) individual readers; (**b**) all readers, senior readers, and junior readers.

**(a) AUC of Each Reader**				
		**Seniority** ** **(Year)**	**AUC**	
			**Without CSF**	**With CSF**
Senior * Readers	Reader 1	25	0.761	0.805
	Reader 2	21	0.786	0.821
	Reader 3	21	0.766	0.804
	Reader 8 ^#^	12	0.807	0.810
	Reader 11 ^##^	7	0.832	0.825
	Reader 12 ^##^	7	0.822	0.817
	Reader 13	15	0.731	0.767
	Reader 14	10	0.614	0.738
	Reader 15	11	0.711	0.805
Junior Readers	Reader 4	5	0.740	0.770
	Reader 5	5	0.609	0.749
	Reader 6	5	0.744	0.753
	Reader 7	2	0.739	0.797
	Reader 9	1	0.514	0.642
	Reader 10	5	0.717	0.781
	Reader 16	3	0.653	0.761
	Reader 17	2	0.633	0.716
	Reader 18	1	0.784	0.810
**(b) Mean AUC**				
	**Without CSF** **(95% CI)**	**With CSF** **(95% CI)**	**Improvement** **(95% CI)**	***p*-Value**
**All Readers**	0.720 (0.661, 0.780)	0.776 (0.708, 0.844)	0.056 (0.002, 0.110)	0.0420
**Senior Readers**	0.759 (0.706, 0.812)	0.799 (0.732, 0.866)	0.040 (−0.014, 0.094)	0.1462
**Junior Readers**	0.681 (0.608, 0.755)	0.753 (0.679, 0.827)	0.072 (0.009, 0.136)	**0.0265**

* Senior Readers: Seniority > 6 years; ** The item indicates the years of the reader been certified as a physician and able to use the ultrasound machine and interpret the sonograms; ^#^ The reader is a board-certified thyroid specialist; ^##^ The reader is a board-certified radiologist; CI: confidence interval; Improvement = With CSF − Without CSF.

## Data Availability

Not applicable.
